# Transposon activation is a major driver in the genome evolution of cultivated olive trees (*Olea europaea* L.)

**DOI:** 10.1002/tpg2.20010

**Published:** 2020-03-27

**Authors:** Jaime Jiménez‐Ruiz, Jorge A. Ramírez‐Tejero, Noé Fernández‐Pozo, María de la O Leyva‐Pérez, Haidong Yan, Raúl de la Rosa, Angjelina Belaj, Eva Montes, Mª Oliva Rodríguez‐Ariza, Francisco Navarro, Juan Bautista Barroso, Carmen R. Beuzón, Victoriano Valpuesta, Aureliano Bombarely, Francisco Luque

**Affiliations:** ^1^ Center for Advanced Studies in Olive Grove and Olive Oils, Department of Experimental Biology University. Jaén Jaén 23071 Spain; ^2^ Plant Cell Biology, Faculty of Biology University of Marburg Marburg Germany; ^3^ School of Plants and Environmental Sciences Virginia Tech Blacksburg VA 24061 USA; ^4^ present address, Department of Bioscience Universita degli Studi di Milano Milan 20133 Italy; ^5^ Centro de Investigación y Formación Agraria de Alameda del Obispo Instituto de Investigación y Formación Agraria y Pesquera (IFAPA) Córdoba Spain; ^6^ Instituto Universitario de Investigación en Arqueología Ibérica University. Jaén Jaén 23071 Spain; ^7^ Departamento de Biología Celular, Genética y Fisiología, Facultad de Ciencias, Instituto de Hortofruticultura Subtropical y Mediterránea Universidad de Málaga ‐ Consejo Superior de Investigaciones Científicas Málaga Spain; ^8^ Departamento de Biología Molecular y Bioquímica, Facultad de Ciencias, Instituto de Hortofruticultura Subtropical y Mediterránea Universidad de Málaga ‐ Consejo Superior de Investigaciones Científicas Málaga Spain

## Abstract

The primary domestication of olive (*Olea europaea* L.) in the Levant dates back to the Neolithic period, around 6,000–5,500 BC, as some archeological remains attest. Cultivated olive trees are reproduced clonally, with sexual crosses being the sporadic events that drive the development of new varieties. In order to determine the genomic changes which have occurred in a modern olive cultivar, the genome of the Picual cultivar, one of the most popular olive varieties, was sequenced. Additional 40 cultivated and 10 wild accessions were re‐sequenced to elucidate the evolution of the olive genome during the domestication process. It was found that the genome of the ‘Picual’ cultivar contains 79,667 gene models, of which 78,079 were protein‐coding genes and 1,588 were tRNA. Population analyses support two independent events in olive domestication, including an early possible genetic bottleneck. Despite genetic bottlenecks, cultivated accessions showed a high genetic diversity driven by the activation of transposable elements (TE). A high TE gene expression was observed in presently cultivated olives, which suggests a current activity of TEs in domesticated olives. Several TEs families were expanded in the last 5,000 or 6,000 years and produced insertions near genes that may have been involved in selected traits during domestication as reproduction, photosynthesis, seed development, and oil production. Therefore, a great genetic variability has been found in cultivated olive as a result of a significant activation of TEs during the domestication process.

**Abbreviations**
DAPC: discriminant analysis of principal components
Ds: dissociation element
GO: Gene Ontology
INDEL: insertion or deletion
LTR: long terminal repeats
ML: maximum likelihood
MCL: maximum composite likelihood
MNP: multiple nucleotide polymorphisms
MYA: million years ago
NJM: neighbor joining method
PCA: principal component analysis
RT: retrotransposon
SNP: single nucleotide polymorphisms
SSR: short sequence repeat
TE: transposable element
VCF: variant call format

## 1 INTRODUCTION

The importance of olive (*Olea europaea* L.) cultivation in the Mediterranean Basin is outstanding. Olive crop has an undisputable social, economic, and agro‐ecological relevance in some countries. Due to its health benefits and economic relevance in Mediterranean countries, olive oil is one of the most important vegetable oils in the world (Conde, Delrot, & Gerós, 2008). Extra virgin olive oil, a natural fruit juice with exceptional nutritional properties, is of particular interest due to its potential benefits for human health (Donaire, Pedrola, de la Rosa, & Llave, 2011). The world demand of extra virgin olive oil is continuously increasing and, among the different varieties, ‘Picual’ variety extra virgin olive oil features exceptional organoleptic properties and high oxidative stability due to its high content of polyphenolic compounds (Gutiérrez, Arnaud, & Garrido, 2001; Talhaoui et al., 2016).

It is considered that olive domestication began around 6,000–5,500 BC (Kaniewski et al., 2012; Newton, Lorre, Sauvage, Ivorra, & Terral, 2014; Zohary & Hopf, 2000) in the Levant. The development of grafting techniques could have been important to facilitate the olive crop spread by human migrations from the Middle East and Central Asia to Western Europe (Juniper & Maberly, 2006). In fact, the study of chloroplast genetic types shows that the most widely cultivated olive these days probably originated in the area of northwest Syria and southeast Turkey (Besnard et al., 2013b). Archeological records of charred olive stones also sustain the fundamental role played by east–west human migrations in the spread of this crop, since the typical shape of Middle East olives appeared in the west around 3,000 BC (Newton et al., 2014).

Cultivated olive trees are vegetative propagated in order to maintain the cultivar characteristics lost upon sexual reproduction. In this regard, individuals of the same variety are clonal plants. Selection of clonal mutations can explain minor genetic differences, but not the large differences found between varieties, so new recombinants produced by sexual reproduction that eventually generates new and improved genotypes is hypothesized to be the way in which new varieties have appeared. This fact, along with the slow growth and long life of these trees, has limited the appearance of new varieties. However, probably around 2,000 olive varieties with a high phenotypic variability have been developed over a low number of sexually reproduced generations. The complex history of ancient civilizations that moved across the Mediterranean renders the task of getting a real picture of the origin of the present varieties difficult. Genetic admixture may have also occurred at early steps of domestication (Besnard et al., 2013a; Diez et al., 2015). Furthermore, local domestication and introgression events may have been involved to produce a complex pattern of cultivar diversification (Diez et al., 2015).

Phenotypes selected by growers drive domestication. The most relevant characters selected in olive domestication were fruit size and oil production. Cultivated olive varieties differ significantly in fruit size, but they are, rather consistently, much larger than wild olive fruits, because the latter, subject to a strong natural selection, remain small as a mechanism of seed dispersal by frugivorous birds (Rey, Gutiérrez, Alcántara, & Valera, 1997). Whether human selection was mainly oriented towards oil production or towards obtaining a large fruit for the elaboration of table olives was highly dependent on the geographical localization. Greek and Roman civilizations were essentially interested in producing olive oil and, therefore, the fruit of cultivated olive varieties in that area is usually smaller than in the upper Middle East, where olives were mainly used as table olives.

The phenotypic diversity associated with domestication processes is generated over shorter periods of time than natural diversity associated with speciation. Whether domestication entirely depends on the initial diversity of original species or whether there are genetic mechanisms to increase the variability during the domestication process should be elucidated. Although no clear answer to this question is yet available, transposable elements (TE) may play a role in generating variability as an important source of natural or human selection. In the case of maize (*Zea mays*), the insertion of the TE *Hopscotch* in an enhancer of the *teosinte branched1* (*tb1*) gene was selected during domestication, but such insertion preceded maize domestication (Studer, Zhao, Ross‐Ibarra, & Doebley, 2011). Similarly, a dramatic amplification of a TE in rice (*Oryza sativa*) has been observed during recent domestication (Naito et al., 2006), and a short interspersed nuclear element has been associated with wolf (*Canis lupus*) domestication (Gray, Sutter, Ostrander, & Wayne, 2010). However, the role of TEs in domestication is yet uncertain.

This study analyzes the putative activation of TEs during olive domestication and its effect on the cultivated olive genome. To that effect, the genome of the *Olea europaea* cultivar Picual has been sequenced and used as the reference genotype. Forty additional genotypes from cultivated olive varieties and 10 wild genotypes have also been sequenced and their phylogenetic relationships have been studied. Furthermore, DNAs from four samples of charred stones found in Roman settlements of the province of Baetica (southern Spain) have been sequenced. The excavations cover most of the occupation from the second and first centuries BC to the fourth century AD and are located in the current provinces of Granada and Jaen (Andalusia), the world's most important olive oil production area. The analysis of these DNA sequences may shed light not only on the origin of the cultivars in the south of Spain but also on human history.

## 2 MATERIALS AND METHODS

### 2.1 Plant material and archaeological samples

Plant samples for this study were obtained from the World Olive Germplasm Collection of the Andalusian Institute of Agricultural and Fisheries Research and Training, and from the wild ex situ repository of the same institution, both located in Córdoba (Andalusia, southern Spain). The cultivated material consists of 36 olive cultivars belonging to a previously established core collection (Belaj et al., 2012), as well as six other economically important cultivars and 10 wild genotypes (Table 4). Archeological samples were obtained from carbonized pit remains from four archaeological excavations dated at the Roman Empire occupation in the Iberian peninsula, from the University Research Institute for Iberian Archeology (Jaen, Spain; Table 4).

#### 2.1.1 DNA extraction and sequencing

DNA was extracted and purified from fresh young leaves with the Illustra Nucleon PhytoPure Genomic DNA Extraction Kit (GE Healthcare) according to the manufacturer's instructions. With regard to archeological remains, due to the poor conservation status of carbonized pits, the CTAB protocol (Kistler, 2012) was modified by pulverizing tissue in liquid nitrogen before adding extraction buffer and cleaning samples twice with cold chloroform and isopropanol.

For the sequencing of the Picual variety genome, DNA was sequenced by shotgun using two next generation sequencing platforms. First, 2 × 125 bp of paired‐end (insert size of 300 bp) and mate‐pairs (insert sizes of 5, 8 y 10 kb) DNA fragments were sequenced by Illumina HiSeq 2500. Second, high molecular weight DNA was used for shotgun PacBio RSII sequencing. Re‐sequencing of the remaining varieties was made by 2 × 150 paired‐end sequencing with Illumina HiSeq 4000. In all cases, sequencing was carried out at the Duke Center for Genomics and Computational Biology (Durham, NC).

**Core Ideas**

- The genome of ‘Picual’ variety is larger than wild genomes and contains a greater number of genes.
- Domestication produced a genetic bottleneck, but olives have recovered a high genetic diversity.
- The driving force of this increase of genetic variability is at least partially due to TEs activation.
- During the domestication process, many TEs families has expanded in cultivated olives.
- TE insertions near genes associated with domestication were found in cultivated olives.

#### 2.1.2 Genome assembly and annotation

Two different approaches were used for assembly optimization. On the one hand, an Illumina‐based assembly was initially performed and then it was re‐scaffolded with PacBio reads. On the other, a PacBio‐based assembly was performed first and then it was re‐scaffolded with Illumina reads.

Illumina reads were first preprocessed using: (a) Fastq‐mcf (Aronesty, 2011) for a quality and length filtering (Q30L50); (b) Musket (Liu, Schroeder, & Schmidt, 2013) for read correction; and (c) FastUniq (Xu et al., 2012) for polymerase chain reaction duplication removal. Processed reads were then assembled with SOAPdenovo2 (Luo et al., 2012) using a wide range of Kmer values (data not shown); better results were obtained for Kmer 95 (in terms of total size, total number of sequences, longest sequences, average sequence length, N50/L50, N90/L90). The gaps for the scaffolds from Kmer 95 assembly were filled with GapCloser (Luo et al., 2012) using Illumina pair‐end reads. Then, the second step was re‐scaffolding using SSPACE‐LongRead and the PacBio reads (Boetzer & Pirovano, 2014), and the third step consisted of gap filling using the PacBio reads and PB‐Jelly (English et al., 2012). Thereafter, Oleur0.1 Illumina‐based draft assembly was obtained.

Genome size was estimated using the Kmer distribution methodology (described in Li et al., 2009). The pair‐end Illumina reads were decomposed in Kmers of 17, 25, and 33 mers and counted using Jellyfish v2.2.6 (Marcais & Kingford, 2011). The different Kmer distributions were analyzed with GenomeScope (Vulture et al., 2017).

PacBio reads were assembled with Canu tool filtering the output contigs with an error rate of .025. Then, the contigs were scaffolded with SSPACE‐standard (Hunt, Newbold, Berriman, & Otto, 2014) and the Illumina Mate Pair libraries. Finally, the gaps were filled with GapCloser using Illumina pair‐end reads. Two extra steps of re‐scaffolding (SSPACE‐LongRead) and a re‐filling (PB‐Jelly) were finally performed to obtain Oleur0.6 PacBio‐based genome draft assembly. Both assemblies were performed in a Very Large Memory node of the Cascades cluster at the Advance Computing Resource center (www.arc.vt.edu) at Virginia Tech.

The structural annotation was performed using MAKER‐P (Campbell et al., 2014) with the default parameters. The annotation was supported by three datasets: (a) transcriptome assembly of olive trees studies such as its response to abiotic (cool; Leyva‐Pérez et al., 2015) and biotic (infection by *Verticillium dahlia*; Jiménez‐Ruiz et al., 2017) stresses and juvenile seedlings (Jiménez‐Ruiz et al., 2015); (b) protein sequence set for the Lamiids downloaded from UniProt database (http://www.uniprot.org/); and (c) repetitive sequences dataset produced by RepeatModeler (http://www.repeatmasker.org/RepeatModeler/), using the *Olea europaea* genome assembly version Oleur061. Genome annotation completeness was evaluated with BUSCO (Simão, Waterhouse, Ioannidis, Kriventseva, & Zdobnov, 2015). The functional annotation was performed using two different methods:

1. The BLAST homology search with TAIR10, GenBank NR (accessed on 8 October 2016) and SwissProt (accessed on 1 November 2016) datasets. Results were filtered for E‐values >10^‐10^.
2. The InterProScan protein domain search including a Gene Ontology (GO) terms association. Both results were integrated using Automated Assignment of Human Readable Descriptions (https://github.com/groupschoof/AHRD) with weights of 100 (Swissport), 75 (TAIR10), and 30 (GenBank NR). All the annotation processes were performed in an Ubuntu server with 64 cores, 128 GB of RAM, and 3 TB hard drive.

### 2.2 Comparison between genome assemblies of wild and Picual accessions

The *O. europaea* var. Picual were mapped to the *O. europaea* ssp. *sylvestris* genome assembly version Oe451 using BlasR Smrtanalysis‐3.0 (Chaison & Tesler, 2012) and NGMLR (Sedlazeck et al., 2018). Sam/Bam files were filtered using Samtools v1.3.1 (Li et al., 2009). Structural variants were analyzed with Sniffles (Sedlazeck et al., 2018). Gene family clustering was performed following the methodology described in the Computational Analysis of gene Family Evolution (CAFE; https://hahnlab.github.io/CAFE/manual.html; Han, Thomas, Lugo‐Martinez, & Hahn, 2013). In brief, 11 species, mostly asterids, were selected for this analysis: *Actinidia chinensis* (version GCA_003024255.1; source NCBI genome), *Arabidopsis thaliana* (version TAIR10; source: The Arabidopsis Information Resource [TAIR]), *Daucus carota* (version GCA_001625215.1; source NCBI genome), *Mimulus guttata* (version GCA_000504015.1; source NCBI genome), *Nicotiana sylvestris* (version GCA_000393655.1; source NCBI genome), *Olea europaea* ssp. *sylvestris* (version GCA_002742605.1/Oe451; source NCBI genome), *Oryza sativa* (version GCA_001433935.1/IRGSP‐1.0; source NCBI genome), *Sesamum indicum* (version GCA_000512975.1; source NCBI genome), *Solanum lycopersicum* (version ITAG3.1; source: Sol Genomics Network), and *Vitis vinifera* (version GCA_000003745.2; source NCBI genome). All the predicted protein datasets were concatenated in a single file, which was used to perform a homology search by BLAST. Columns 1, 2, and 11 from the tabular output were selected and used as input for the maximum composite likelihood (MCL) with an inflation parameter of three. The MCL output was parsed with the CAFE script cafetutorial_mcl2rawcafe.py. The output was filtered with a simple Bash script using cut and awk, and the family size was compared for Oe451 and Oleur061 assemblies.

Genome heterozygosity was calculated by mapping the Illumina pair‐end reads back to both assemblies (Oleur061 and Oe451) with BWA v0.7.15 (Li & Durbin, 2009). Sam/Bam files were filtered and sorted with Samtools v1.3.1 (Han et al., 2013) and variants were called using FreeBayes v1.1.0 (Garrison & Marth, 2012) with a minimum read coverage of 10 and a minimum mapping quality of 20. Heterozygous variants were selected with grep “0/1” and then counted with the GenoToolBox script Vcf2CountingBins (https://github.com/aubombarely/GenoToolBox/) with a bin size of 10 Kb. The estimation of the whole genome duplications was performed with CoGe SyMap tool (Lyons, Pederson, Kane, & Freeling, 2008) and topGO v3.3 R package (Alexa & Rahnenfuhrer, 2016) was used for gene set enrichment analysis.

#### 2.2.1 Phylogenetic and population analyses

The Illumina reads of the re‐sequenced accessions were processed as described in Materials and Methods, section “Genome assembly and annotation,” except in archaeological olive pit remains reads, where the 12 bp of the 5′ and 3′ extremes were removed with Seqtk v1.2. Reads were mapped and variants were called as described in Material and Methods, section “Comparison between genome assemblies of wild and ‘Picual’ accessions.” Variants were filtered for: (a) no missing data, (b) only biallelic, (c) one variant each 10 Kb, and (d) minimum variant quality of 30 with VCFtools version 0.1.15 (Danecek et al., 2011).

The distance‐tree analysis was performed using the neighbor joining method (NJM) and maximum likelihood (ML) methods. For the NJM, the aboot functions with sample = 100 and cutoff = 50 and the plot.tree from the Ape v5.2 R package were used (Paradis & Schliep, 2019). In relation to the ML method, first, the variant call format (VCF) file was converted into Phylip format using the VCF2phy script (https://github.com/CoBiG2/RAD_Tools/blob/master/VCF2phy.py). Then, the ML method was applied through the T‐REX web server (Boc, Diallo, & Makarenkov, 2012) (http://www.trex.uqam.ca/) with the PhyML tool (Guindon & Gascuel, 2003) and supported by a nonparametric bootstrap analysis of 100 replicates. The nucleotide substitution model was determined by MODELTEST (Posada & Crandall, 1998) in its web implementation (http://hiv.lanl.gov/content/sequence/findmodel/findmodel.html) and the general time reversible model was selected.

The different olive accessions were clustered using two different tools: fastStructure (Raj, Stephens, & Pritchard, 2014) and discriminant analysis of principle components (DAPC) from the Ape v5.2 R package (Paradis & Schliep, 2019); fastStructure was run from K = 1 to K = 20 with the default parameters. The optimal K was calculated using the ChooseK.py script from the same package. The DAPC was performed with the Ape v5.2 R package with default parameters. Population genetic parameters were calculated with the Ape R package.

#### 2.2.2 Transposon landscape analysis

The TE‐ long terminal repeats (LTRs) identification in both the ‘Picual’ accession (Oleur061) and the wild olive (Oe451) genome assemblies was based on LTRfinder (Xu & Wang, 2007) and LTRhavest (Ellinghaus, Kurtz, & Willhoeft, 2008). The results from LTRfinder and LTRhavest were combined for the Picual cultivar reference and the wild reference (Unver et al., 2017), obtaining 38,171 TE‐LTR candidates and 24,400 candidates, respectively. Parameters used were: LTRfinder ‘‐D 20000 ‐d 1000 ‐L 5000 ‐l 100 ‐p 20 ‐C ‐m 0.85’ and LTRharvest ‘‐seed 30 ‐minlenltr 100 ‐maxlenltr 5000 ‐mindistltr 1000 ‐maxdistltr 20000 ‐similar 85 ‐mintsd 4 ‐maxtsd 20 ‐motif tgca ‐motifmis 1 ‐vic 60 ‐xdrop 5 ‐mat 2 ‐mis ‐2 ‐ins ‐3 ‐del ‐3’. All the candidates were annotated for PfamA domains (Sonnhammer, Eddy, & Durbin, 1997) with Hmmer3 (Eddy, 2009), and then classified according to the following criteria: (a) Superfamily Gypsy (code RLG) was considered when protein domain order was gag‐RT/RH‐INT; (b) Superfamily Copia (code RLC) was considered when protein domain order was gag‐INT‐RT/RH; (c) Superfamily unknown (code RLX) was considered when the LTR retrotransposons (RTs) did not contain any coding regions for their internal domain (Wicker et al., 2007). A sequences alignment between 5′ and 3′ LTRs was performed with MUSCLE (Edgar, 2004). Then, the nucleotide variations (λ) in 5′ and 3′ of the LTR‐RTs were calculated. The DNA substitution rates (*K*) were calculated by *K* = − 0.75 ln (1 − 4 λ / 3). The insert time of LTR‐RTs was estimated with the formula *T* = *K*/2*r* (*r* = 1.3 × 10^−8^ per site and per year; Ma & Bennetzen, 2004).

Transposons in the assembly Oleur061 were searched by combining de novo‐ and homology‐based approaches. For the de novo approach, RepeatModeler (http://www.repeatmasker.org/RepeatModeler.html; Smit et al., 2014) was used. For the homology‐based approach, RepeatMasker (http://www.repeatmasker.org, version 3.3.0) and Repbase TE library (Smit et al., 2017) were used. Then, the TEs from these two approaches were combined and Cd‐hit (Fu, Niu, Zhu, Wu, & Li, 2012) was used to remove the redundant TEs with default parameters. Two software including TE‐locate (Platzer, Nizhynska, & Long, 2012) and TEMP (Zhuang, Wang, Theurkauf, & Weng, 2014) in the McClintock pipeline (Nelson, Linheiro, & Bergman, 2017) were used to detect TE insertions in the re‐sequenced olive accessions. The TE insertion numbers of three population groups (Cluster1‐Wild, Cluster2‐Cul1 and Cluster3‐Cul2) were calculated and compared with each other via T‐test (< .05 as significant difference).

The TE location information was obtained from McClintock pipeline (Nelson et al., 2017). The TE loci from the reference genotype were used as reference TEs (RefTEs), and set 500‐bp flanking regions as well as the body of RefTEs as a reference ranges (RefRGs; the range length of each RefRG is 1 kb added to the body length of the RefTE). For one genotype in a population, if one RefRG contains TEs, this genotype will be regarded as having a RefTE. The number of genotypes with or without the RefTEs was calculated for the three populations (Cluster1‐Wild, Cluster2‐Cul1 and Cluster3‐Cul2). The Fisher's exact test was used to identify the candidate RefTEs and they were classified based on the following criteria: c1_c2_ls_w is defined as RefTEs that were enriched into the wild population when compared with Cul1 and Cul2; c1_ls_w and c2_ls_w are defined as RefTEs that were enriched into the Wild population but not in Cul1 and Cul2, respectively; c2_eq_c1_gt_w is defined as RefTEs that were enriched into the Cul1 and Cul2 but not in the Wild population, and Cul1 and Cul2 have similar RefTEs; c2_gt_c1_gt_w is defined as similar to c2_eq_c1_gt_w, but has more RefTEs in Cul2 than in Cul1; c2_gt_c1_eq_w is defined as RefTEs which are only enriched in the Cul2 but not in Cul1 and Wild populations. For the identification of potential genes influenced by the RefTEs, a flanking region (3,000 bp) was set to detect the surrounding genes with the help of the gene annotation general feature format (GFF) file.

#### 2.2.3 RNA‐Seq analysis

The RNA‐Seq expression data were analyzed with the ArrayStar Lasergene 15 package, using a 95% false discovery rate, whereby the reference transcriptomes of Picual genes and TE elements were obtained. RepeatModeler identified 2,370,444 interspersed repeats in the raw genome, however, only 339,518 were catalogued by RepeatMasker as putative transposable elements in plants. Further, 287,453 elements longer than 100 bp were filtered. The RNA‐Seq reads publicly available (Leyva‐Pérez et al., 2015; Leyva‐Pérez et al., 2018) of ‘Picual’ and ‘Frantoio’ root and leaf (two replicates per sample) were mapped using Bowtie2 (Langmead & Salzberg, 2012) and Rsem (Li & Dewey, 2011) in order to obtain a reference transcriptome of TE composed by 50,326 expressed transcripts. The TEs expressed in the different cultivars (minimum 2 reads per kb and millions of reads) were quantified, categorized and grouped.

## 3 RESULTS AND DISCUSSION

### 3.1 Genome assembly, annotation, and quality evaluation

The assembly of *Olea europaea* ‘Picual’ genome was based on the integration of different sequencing technologies through different rounds of assembly, scaffolding and gap filling. The two sequencing technologies used in this experiment were Illumina short reads and PacBio long reads. One paired‐end (with an insert size of ∼300 bp) and three mate‐pair (with an insert size of ∼5 Kb, ∼8Kb and ∼10 Kb) Illumina libraries were sequenced delivering 623.28 Gb and 363.59 Gb, respectively. Paired‐end reads were used to estimate the genome size and the heterozygosity using the Kmer distribution approach (described in Li et al., 2010). The genome size ranged from 1.63 to 1.81 Gb. The estimated heterozygosity was around 2.02%. Additionally, 75 PacBio Smart Cells were sequenced from the same samples yielding 68.30 Gb of long reads with an average size 12.06 kb.

Two different approaches were used to assemble *Olea europaea* cv. Picual genome: (a) an Illumina‐based assembly improved with PacBio reads using SSPACE‐LongRead to re‐scaffold the contigs and PB‐Jelly to fill the gaps (named “Oleur011”); and (b) a PacBio‐based assembly improved with Illumina reads using SSPACE‐standard to scaffold the PacBio contigs and GapCloser to fill the gaps (named “Oleur061”). Both assemblies were evaluated based on their assembly statistics and RNA‐Seq mapping stats, as summarized in Table 1. Oleur061 assembly was selected as a canonical draft for further steps.

**TABLE 1:** Assembly stats of the sequenced olive genomes

Assembly stats	Oleur0.1.4 (Illumina based)	Oleur0.6.1 (PacBio based)	OE6A (Cruz et al., 2016)	Oe451 (Unver et al., 2017)
Total assembly size, Gb	2.48	1.68	1.32	1.14
Total number of sequences	42,316	8,718	11,038	41,256
Total gap size, Mb	314.19	9.00	53.97	110.81
Longest sequence, Mb	3.82	4.14	2.58	46.03
Average sequence length, Kb	58.62	192.24	119.46	27.69
N90, number of sequences	16,115	4,475	3,099	3,410
L90, Kb	26.26	87.94	110.96	22.60
N50, number of sequences	3,099	1,145	901	23
L50, Kb	197.15	410.80	443.10	12,567.91
Sequences >1 Mb	111	177	129	39
% BUSCO complete	89.7	93.5	94.3	87.3
% BUSCO duplicated	51.8	50.4	23.9	13.6
% BUSCO fragmented	2.4	0.8	0.7	2.0
% BUSCO missing	7.9	5.7	5.0	10.7
Map % SRR6003535^a^ RNA‐Seq	94.36	95.55	92.97	84.03
Map % ERR1406351^b^ RNA‐Seq	93.88	94.94	95.40	84.46
Map % SRR8654633^c^ RNA‐Seq	92.19	93.24	91.30	84.36

Three public Illumina RNA‐Seq experiments were used to evaluate the gene space captured by the assembly:

a SRR6003535 (*Olea europaea* ‘Arbequina’ seedling development).

b ERR1406351 (*Olea europaea* ‘Farga’ immature olives).

c SRR8654633 (*Olea europaea* var. *sylvestris*).

The genome assembly was verified by mapping back PacBio reads with BlasR and checking the coverage of each of the scaffolds. The average mapping coverage was 16X with 95% of the assembly covered by five reads or more. A total of 355 scaffolds (2.81 Mb) covered with less than five reads were removed from the assembly. The screening of nonplant contaminants did not reveal major contaminations, except for 101 small scaffolds (<10 Kb) that were also removed from the analysis. Additionally, the Pseudohaploid tool (https://github.com/schatzlab/pseudohaploid) was used to mark possible uncollapsed haplocontigs produced during the assembly. Nevertheless, no contigs were identified as redundant haplocontigs.

The genome annotation produced 79,667 gene models, of which 78,079 were protein coding genes and 1,588 were tRNA. The average number of exons per gene model was 5.04, which falls in the range between *Arabidopsis thaliana* (6.10) and *Solanum lycopersicum* (4.61). Average mRNA and protein sizes were 1,152 bp and 316 amino acids respectively. The BUSCO analysis of the annotation indicated that although the missing BUSCO genes percentage (92 models, 6.3%) is similar to the BUSCO analysis on the genome (83 models, 5.7%), the percentage of fragmented genes (80 models, 5.6%) is higher (11 models, 0.8%; Table 1).

The repetitive landscape was similar to other plant species in global terms. Accordingly, 59% of the genome assembly consisted of repetitive elements, being in the same range than previously sequenced olive genomes, 51% for *Olea europaea* var. *sylvestris* (Unver et al., 2017) and 63% for *O. europaea* ‘Farga’ (Cruz et al., 2016). Long terminal repeat elements were the most abundant repetitive elements accounting for 29% of the assembly, followed by unidentified repetitive elements (22%), simple repeats (3%), DNA transposons (3%), and long interspersed nuclear elements (1%) (Supplemental Table S1 for a more detailed description of the repeats).

The comparison of the assembly stats and completeness of Oleur061 with previously published genomes (Cruz et al., 2016; Unver et al., 2017) revealed not only an assembly size that is close (1.68 Gb) to the expected genome size (1.81 Gb) but also an assembly that has captured a higher proportion of the gene space (up to 95.55% of mapped RNA‐Seq reads and 93.5% of complete BUSCO models) and its complexity (50.4% of duplicated BUSCO models; Table 1). As a result, 21,730 and 27,392 more gene models were annotated compared to previously published genomes (Cruz et al., 2016; Unver et al., 2017).

### 3.2 Comparison between genome assemblies of wild and ‘Picual’ accessions

As pointed out above, the ‘Picual’ accession showed 27,392 more gene models than the wild accession. In order to elucidate its source and some other differences, two approaches were applied.

First, the PacBio processed reads were mapped to the wild olive genome reference Oeuropaea451 (Oe451). A total of 13.81 millions of reads was mapped to the Oe451 representing 99.8% of the PacBio reads used in our assembly. The assembly Oe451 showed 110.81 Mb of gaps. The PacBio read mapping left 114.37 Mb not covered in the assembly Oe451. The analysis of the structural variants revealed 44,750 translocations between Oe451 (*Olea europaea* var. *sylvestris*) and *Olea europaea* ‘Picual’. The number of translocations ranged from 422 (Chromosome 23) to 2010 (Chromosome 10; Supplemental Table S2).

Second, the annotated genes were grouped by families and the gene ratio between Oleur061 (*Olea europaea* ‘Picual’) and Oe451 (*Olea europaea* var. *sylvestris*) assemblies was subsequently analyzed. A high number of gene families (3,436) presented twice as many genes in Oleur061 as in Oe451. A total of 1,076 and 437 gene families showed three and four times as many genes in Oleur061 as Oe451 respectively. Nevertheless, only 134 families showed two or more gene models in Oe451 than in Oleur061. These results are in line with the trend found when the total number of annotated protein coding genes for Oleur061 (78,079) and Oe451 (50,684) was compared. There are four possible reasons for this result: (a) Oleur061 annotation has an elevated number of fragmented genes that have been annotated as complete genes; (b) Oleur061 assembly has a high number of uncollapsed haplotype regions that double the number of genes; (c) *O. europaea* ‘Picual’ genome contains a large number of fragment DNA duplications compared to the sequenced *O. europaea* var. *sylvestris;* and (d) Oe451 assembly has a significant number of collapsed paralog genes. The first reason can be ruled out based on the average number of exons per gene (5.04) and mRNA length (1,152 bp) for Oleur061. They were similar to Oe451 (4.61 and 1,040 bp). To study the number of collapsed regions, the number of heterozygous variants per gene was analyzed after remapping their own reads. A low number of variants can be an indicative of uncollapsed regions. The calculated heterozygosity per bin (with a size 10 Kb) was 0.7% ± 2.8%. Only 15.6% of the bins presented a heterozygosity <0.1%. The estimated genome size and heterozygosity using the Kmer distribution were 1.81 Gb and 2.02% respectively. In light of these results, there is strong evidence that most of the two haplotypes have been collapsed in a consensus sequence and it is unlikely that the higher number of genes in Oleur061 assembly is caused by the independent assembly of both haplotypes. Additionally, the Pseudohaploid tool was run and no haploid contig redundancy was detected (see previous section). The estimated genome size for *O. europaea* ‘Picual’ ranges from 1.63 to 1.81 Gb using Kmer analysis, and Oleur061 assembly was 1.68 Gb. Since previously described *O. europaea* genome sizes ranged from 1.65 Gb for ‘Manzanilla’ variety (Besnard et al., 2008) to 2.21 Gb for Leccino variety (Rugini, Pannelli, Ceccarelli, & Muganu, 1996), but there is not strong evidence that ‘Picual’ could be a polyploid variety compared to other *O. europaea* varieties. Nevertheless, the publicly available assembly of Oe451 has a total size of 1.14 Gb (NCBI accessions GCF_002742605.1) representing a minimum of 0.32 Gb below the estimated genome size (Unver et al., 2017). In order to know whether the wild genome is smaller than the ‘Picual’ variety genome or whether there is a high number of collapsed paralogs in the Oe451 wild genome assembly, Illumina reads of four re‐sequenced wild genomes (accessions Wild05, Wild06, Wild07, and Wild09) as well as a ‘Picual’ wild genome were mapped on the Oleur061 assembly. The result was that 175.4 ± 25.3 Mb and 8941 ± 805 genes of Oleur061 were uncovered by the wild sequence reads (Supplemental Table S3). This result suggests that the Picual variety genome may be larger and may contain more genes than wild genomes. Additionally, the fact that the differences of size and gene number of the re‐sequenced wild genomes were approximately half as many as the differences found in Oe451 implies that this assembly may have a number of collapsed paralogs introducing a bias in the number of annotated genes. Unfortunately, the DNA raw reads used for Oe451 could not be retrieved from any of the public repositories to test this hypothesis through the analysis of the variant density using *O. europaea* var. *sylvestris* reads. The bigger size of the ‘Picual’ variety genome may be explained by the fact that it contains a large number of fragment DNA duplications, which are apparently very recent.

**FIGURE 1:**
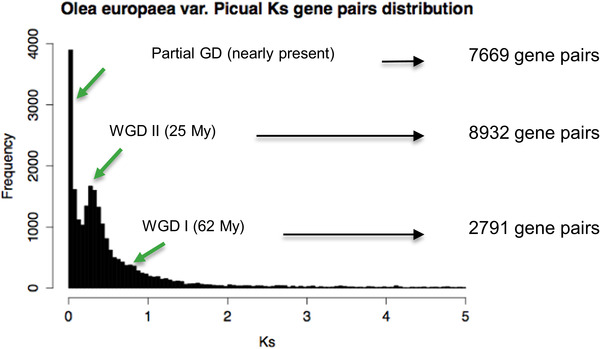
Gene duplication (GD) analysis of Picual genome. Gene duplication analysis showing putative partial or whole genome duplications events (WGD). Ks, synonymous substitution rate

As described for the wild genome (Unver et al., 2017), signs of two whole genome duplication events can be detected in the cultivated ‘Picual’ genome, but in the ‘Picual’ genome it was also found a very recent partial genome duplication event (Figure 1). Diploid *Olea* species have been described as 2*n* = 2*x* = 46. The same number of chromosomes has been described in the close related genus *Fraxinus*, *Osmanthus*, and *Phillyrea* but not in the genus *Jasminum* (2*n* = 2*x* = 26), *Fontanesia* (2*n* = 2*x* = 26) and *Forsythia* (2*n* = 2*x* = 28) from the Oleaceae family (Taylor, 1945). It has been hypothesized that modern Oleaceae species such as *Olea* and *Fraxinus* come from an ancestral allotetraploid produced by the hybridization of an ancestral *Fontanesia* related species with 12 pairs of chromosomes and an ancestral *Jasminum‐Forsythia* species with 11 pairs of chromosomes (Taylor, 1945) between 34 and 65 million years ago (MYA). Previously published *Olea europaea* genomes (Cruz et al., 2016; Unver et al., 2017), as well as the *Fraxinus excelsior* genome (Sollars et al., 2017), supported this hypothesis with a peak around 0.25 in the estimation synonymous substitution rate (Ks) for gene pairs. The estimation of the whole genome duplication event (∼28 MYA) is in line with the estimated age of speciation of the *Olea‐Fraxinus* ancestor. Using a synonymous substitution rate of 6/billion years (estimated in Arabidopsis), a Ks = 0.75, corresponding to 62 MYA, a Ks = 0.3, which corresponds to 25 MYA, and an extra 0.01< Ks <0.1, which represents a very recent event of fragment DNA duplications, were found in ‘Picual’ (Figure 1). After identifying a partial duplication of the ‘Picual’ variety genome, the type of genes included in this duplication was determined by searching for genes covered by reads in ‘Picual’, and with no reads in the four wild re‐sequenced genomes previously analyzed for the genome size. It was found that 3,386 genes fulfilled these requirements. The Biological Process GO terms of ‘Picual’ extra genes with functional annotation are generally related to transcription, transport, metabolism (including lipid metabolism), development, and defense. In addition, the methylation GO term among these genes is present in ‘Picual’ but absent in the four wild accessions analyzed (Supplemental Figure S1A). The Molecular Function GO terms include many transcription factors as well as many genes related to metal binding activity, nucleotide and DNA binding, and different activities associated to an active metabolism (Supplemental Figure S1B).

#### 3.2.1 Genetic structure of domesticated olive trees

Samples of 40 varieties and 10 wild olive trees were re‐sequenced using Illumina paired‐end technology to elucidate the evolution of olive genome during the domestication process (Table 2). The reads of re‐sequenced genomes were aligned to Oleur061 genome reference. The variant calling and filtering delivered a total of 13,435,081 single nucleotide polymorphisms (SNPs). A second filtering to remove nonbiallelic and variants with a distance of less than 1 Kb was performed delivering 148,611 SNPs.

**TABLE 2:** *Olea europaea* re‐sequenced accessions via principle component analysis (PCA)

Collection	Variety	Origin	PCA cluster
World Olive Germplasm Collection	Abbadi Abou Gabra‐842	Syria	3
	Abou Satl Mohazam	Syria	3
	Abou Kanani	Syria	3
	Arbequina	Spain	2
	Barnea	Israel	2
	Barri	Syria	3
	Chemlal de Kabylie	Algeria	2
	Dokkar	Tunisia	1
	Forastera de Tortosa	Spain	2
	Frantoio	Italy	2
	Grappolo	Italy	2
	Hojiblanca	Spain	3
	Fishomi	Iran	3
	Jabali	Syria	3
	Kalamon	Greece	3
	Klon‐14‐1812	Albania	2
	Koroneiki	Greece	2
	Leccino	Italy	2
	Lechín de Sevilla	Spain	3
	Lianolia Kerkyras	Greece	2
	Llumeta	Spain	2
	Maarri	Syria	3
	Manzanilla de Sevilla	Spain	3
	Manzanillera de Huercal Overa	Spain	2
	Mari	Iran	3
	Mastoidis	Greece	2
	Mavreya	Greece	2
	Majhol‐1013	Syria	3
	Majhol‐152	Syria	3
	Menya	Spain	2
	Morrut	Spain	3
	Myrtolia	Greece	2
	Ocal	Spain	3
	Picudo	Spain	3
	Piñonera	Spain	2
	Royal	Spain	3
	Temprano	Spain	3
	Uslu	Turkey	3
	Verdial de Velez‐Malaga‐1	Spain	3
	Zarza	Spain	3
Wild Trees	W1R198 (ssp. *europaea*)	Croatia	2
	W2R74 (ssp. *europaea*)	Spain	1
	W3R78 (subsp. *europaea*)	Spain (Menorca island)	1
	W4R183 (ssp. *europaea*)	Spain	2
	W5R121 (subsp. *guanchica*)	Spain (Gran Canaria island)	1
	W6R1048 (ssp. *guanchica*)	Spain (Tenerife island)	1
	W7R224 (ssp. *europaea*)	Morocco	1
	W8R225 (ssp. *europaea*)	Morocco	2
	W9R302 (ssp. *europaea*)	Albania	1
	W11R37 (ssp. *europaea*)	Spain	1

**FIGURE 2:**
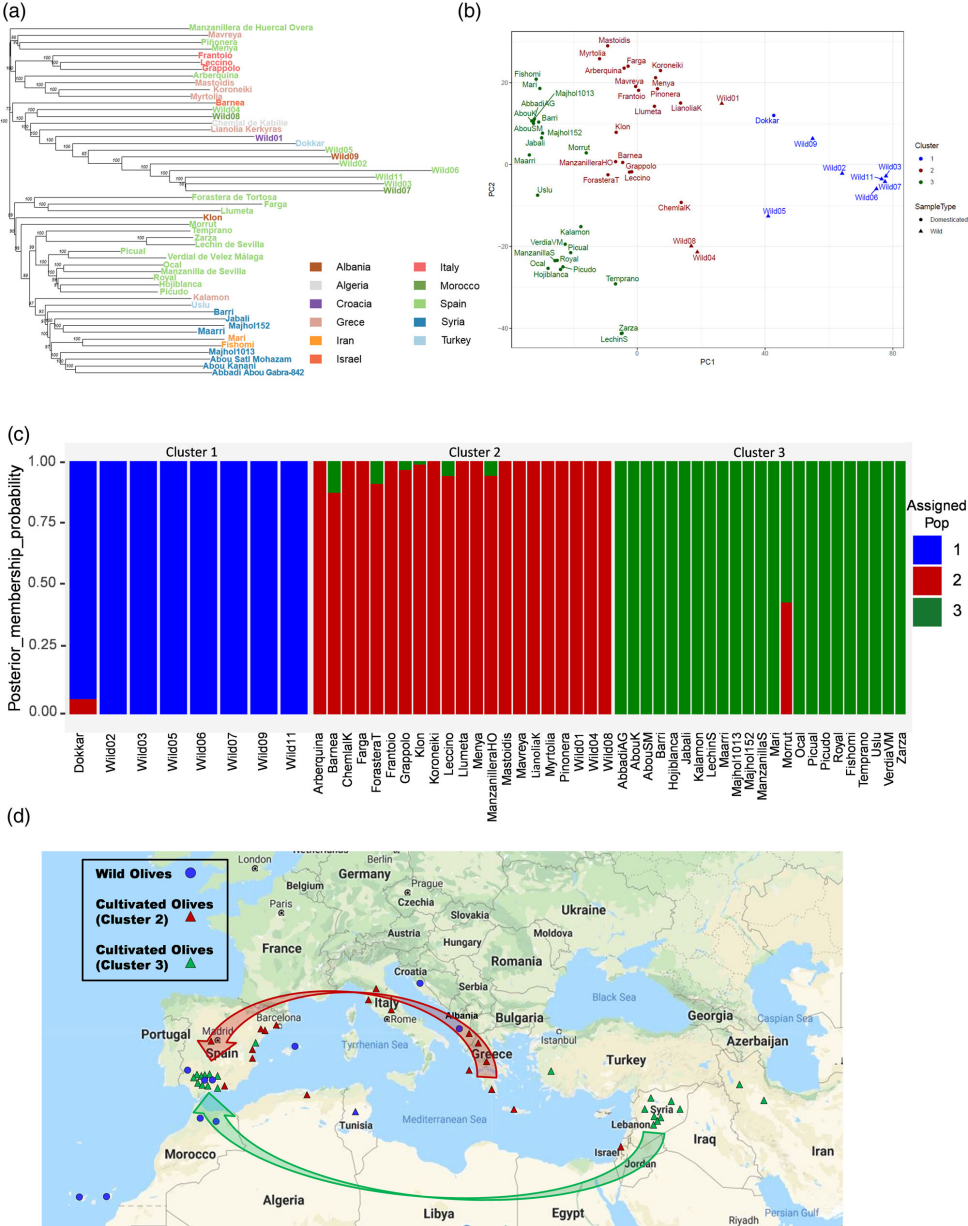
Phylogeny, principal components, and geographic distribution of sequenced cultivated and wild olives. (a) Rooted by outgroup Maximum Likelihood phylogenetic tree (hierarchical view) of wild and cultivated olives. (b) Principal component analysis (PCA). (c) Discriminant analysis of principal components (DAPC). (d) Geographic distribution of wild and cultivated olives. Blue circles represent wild olives, red triangles represent cultivated olives Cluster 2, and green triangles represent cultivated olives Cluster 3. cultivated ‘Dokkar’ variety is represented by a blue triangle. Red arrow represents the north route for olive cultivation spreading, and green arrow the south route

A distance tree was constructed in order to elucidate the genetic relationship between the different genotypes under study (51) including the cultivar ‘Farga’ (Cruz et al., 2016) as well (Figure 2a). The tree revealed two major well supported groups (A–B), with each of them being divided into two subgroups A1, A2 and B1, B2, respectively. The subgroup A1 consisted of wild genotypes from Spain, Morocco, and Croatia and four cultivars: ‘Dokkar’ (Tunisia), ‘Lianolia Kerkyras’ (Greece), ‘Chemlal de Kabilye’ (Algeria), and ‘Barnea’ (Israel). The subgroup A2 consisted of cultivars from Greece (‘Mavreya’, ‘Mastoidis’, and ‘Myrtolia’, Italy (‘Frantoio’ and ‘Leccino’), and Spain (‘Arbequina’, Piñonera’, ‘Menya’, and ‘Manzanillera de Huercal Overa’. While within the group B, eight cultivars from Syria, two from Iran, and the Greek cultivar ‘Kalamon’ clustered together into the subgroup B1; the subgroup B2 included mostly Spanish cultivars, ‘Forastera de Tortosa’, ‘Farga’, and ‘Morrut’ being the ones that clustered farthest from the rest. ‘Klon 14’ from Albania was also included in this subgroup The relationship between the different accessions was also explored using a principal component analysis (PCA; Figure 2b) and DAPC (Figure 2c). The DAPC suggested that the samples could be mainly clustered into two groups (wilds and cultivated), closely followed by the possibility of clustering the samples into three groups (Clusters 1, 2, and 3), including Cluster 1, with seven wild genotypes and ‘Dokkar’ (subgroup A1) as suggested by the distance tree. The DAPC clustering also classified some wild accessions (Wild01, Wild04, and Wild08) and the cultivars ‘Lianolia Kerkyras’, ‘Chemlal K.’, and ‘Barnea’ as part of Cluster 2, rather than as part of Group A1, as suggested by the distance tree. Almost no admixture was detected in the samples, except for Morrut (Spain, Cluster 3) with some contribution of Cluster 2. A population structure analysis using Structure showed similar results. Population clustering inferred from the distance tree and the DAPC showed some association between population structure and fruit size. Cluster 1 members, which are mostly wild accessions and the Dokkar cultivar, are distinguished by small fruits with fruit flesh weights below 0.70 g. In this cluster, Wilds 5 and 6 belongs to the *guanchica* subspecies, but they cluster with the *europaea* subspecies wild trees, indicating a very close genetic relationship between both subspecies, undistinguishable in this analysis. Two main genetic pools of wild olive trees have been described in the Mediterranean basin using eight genomic short sequence repeats (SSRs; Belaj et al., 2007), and three lineages according to a plastid DNA study (Besnard et al., 2013b). In this work, seven of 10 wild trees are found to be grouped together in Cluster 1 and the other three belongs to the Cluster 2 and might belong to a second genetic pool of wild trees closer to the Cluster 2 cultivated varieties. Cluster 2 members, that is, three wild accessions and cultivars from Greece, Italy, and northeaster Spain, are characterized by small‐medium size fruits with fruit flesh weights between 0.61 g (‘Koroneiki’, Greece) and 3.24 g (‘Barnea’, Israel). Cluster 3 members are cultivars with generally bigger fruit sizes (from 3.51 to 7.17 g) from Syria, Turkey, and Southern Spain, with the exception of eight accessions from Spain (‘Zarza’, ‘Verdial de Velez Málaga’, ‘Lechin de Sevilla’), Syria (‘Majhol152’, ‘Barri’, ‘Maarri’), Albania (‘klon’), and Turkey (‘Uslu’). Diez et al., 2015 made a large study of the Mediterranean cultivated olives using 25 SSRs and grouped them into three clusters and roughly, the Cluster 2 of our work corresponds to the Q2 of their study and Cluster 3 with their Q1 and Q3 groups. In fact, our Cluster 3 could be subdivided in two subclusters in both the phylogenetic tree and the PCA that are basically coincident with the Q1 and Q3 of Diez et al., 2015. Thus, the large genome analysis of our shorter list of cultivated tree accessions is consistent with that larger SSRs analysis. The clustering of the cultivated genotypes into different groups (Figure 2d) has a strong geographical component, but at the same time it may indicate a possible phenotypic selection for traits such as fruit size (Besnard et al., 2013b; Belaj et al., 2007; 2012; Diez et al., 2015). The negative values of Tajima's D for the domesticated groups (−0.1161 and −0.3958 for cultivated olives of Clusters 2 and 3 respectively; Table 3) support the hypothesis of a strong selection of specific individuals during domestication as opposed to the lack of rare alleles in wild accessions (0.2852). Estimators of nucleotide diversity (π and θ_w_; Table 3) do not show important differences between groups, which suggests two possible scenarios. In the first one, the domesticated groups have recovered the genetic diversity lost after the possible genetic bottleneck caused by strong selection. In the second, due limitations in the sampling, the analyzed wild accessions do not represent the genetic diversity contained in the ancestral wild populations introducing some biases in the estimation of the real nucleotide diversity driving to the absence of important differences between groups.

**TABLE 3:** Estimates of genetic structure and diversity

Group	*n*	Tajima's D	H^a^	π	θ*_w_*	FST
Cluster 1, Wild	8	0.2852	0.8141	13.193	12.670	0.2200
Cluster 2, Domesticated	21	−0.1161	0.8168	11.836	10.640	0.1310
Cluster 3, Domesticated	22	−0.3958	0.7773	9.196	12.208	0.1945

a H, haplotype diversity; π, nucleotide diversity; θ*_w_*, Theta Watterson; FST, nucleotide fixation index.

The geographical clustering of the majority of the olive cultivars under study and the clear separation between southern and northeastern Spanish olive cultivars may indicate a local selection of olive cultivars as well as a possible expansion of olive growing from the East to the West Mediterranean Basin along the South and the North Mediterranean coasts (Figure 2d).

Additionally, four samples of ancient DNA obtained from seed remains found in four archaeological excavations going back to the Roman Empire were dated using radiocarbon analysis and sequenced (Supplemental Figure S2; Table 4). The DNA obtained from each sample was negligible, but the presence of olive DNA was confirmed by polymerase chain reaction. Most of the sequenced reads corresponded to fungi and bacteria, and the olive‐DNA containing region ranged only from 0.09 to 0.22%, corresponding to 266,498 to 648,952 reads. The reads covered the 0.025, 0.036, 0.028, and 0.096% of the olive genome, corresponding to 35,097, 50,488, 38,668, and 134,096 informative DNA markers (SNPs; multiple nucleotide polymorphisms, MNPs; or insertions or deletions, INDELs). The medium size and the wrinkled surface of the ancient seed remains were indicative of being representatives of domesticated olive varieties. The DNA sequences from the four samples proved to be quite similar, suggesting that during those 5–6 centuries there was a continuum in the cultivated varieties used to produce olive oil. As the DNA obtained from the samples was very scanty and damaged, especially at the ends, 12 bp were removed from both ends and then compared with the rest of genomes by using the ML phylogenetic tree with PhyML (Supplemental Figure S2). The four samples showed to be closer to the present eastern Spanish varieties of Cluster 2, especially to ‘Farga’, followed by ‘Forastera’, ‘Llumeta’, and ‘Menya’. As olive domestication was likely initiated in the east of the Mediterranean Basin, the former indicates that these ancient samples could have been originated by local selection. However, possible crossings with cultivars from other Mediterranean areas during the Roman occupation or other civilizations that has passed cannot be excluded. During the colonial period of the first millennium BC, the Greek colonization expanded across the northwestern Mediterranean Sea while Phoenicians mainly dominated the southeastern Mediterranean area (Figure 2d). Several centuries later, the Islamic expansion from the eighth to the 15th century AD overlapped with the Phoenician colonization. In view thereof, determining the time in which olive cultivation was introduced from the eastern Mediterranean area is essential to know the history and origin of Andalusian olive tree varieties. The sequencing of archeological samples from Roman settlements showed that the samples were closely related to ‘Farga’ and other Cluster 2 varieties from eastern Spain (Supplemental Figure S2). Therefore, in that period the olive cultivars in Andalusia probably came from the north route brought by the Romans and belong to the Cluster 2. However, at present in Andalusia, most cultivars correspond to the Cluster 3 and they probably came from the south route brought by the Arabs.

**TABLE 4:** Archeological remains sequenced samples. Samples of ancient DNA obtained from charred bones found in Roman excavations in Jaen and Granada (South of Spain). Age of the samples determined by radiocarbon analysis

Archaeological sites	Source	Time	Reference
The Village of Gabia, Las Gabias, Granada	Samples from the ground of an oil mill	251–397 AD	Rodríguez‐Ariza and Montes, 2010; Montes, 2014
Marroquíes Bajos Roman oil factory, Jaén	Industrial complex	128–258 AD	Montes, 2014; Serrano, 2004; Serrano and Cano, 1999
Cerro de la Atalaya, La Higuera, Jaén	Crossing and production center	191–38 BC	Barba, Fernández, and Torres, 2016
The Roman Iberian city of Cástulo, Linares, Jaén	Urban and habitation context	251–397 AD	Pérez, 2014; Ceprián, Expósito, Soto, and López, 2016

#### 3.2.2 Evolution of transposon landscape

Transposable elements are known to be a factor in phenotypic diversity, gene expression changes, and genomic instability. When compared with the wild genome, the high complexity of the cultivated olive genome—which displays a large number of chromosomal rearrangements such as duplications and translocations—may be the result of TE activity. To test this hypothesis, we have performed three different analysis described below.

**FIGURE 3:**
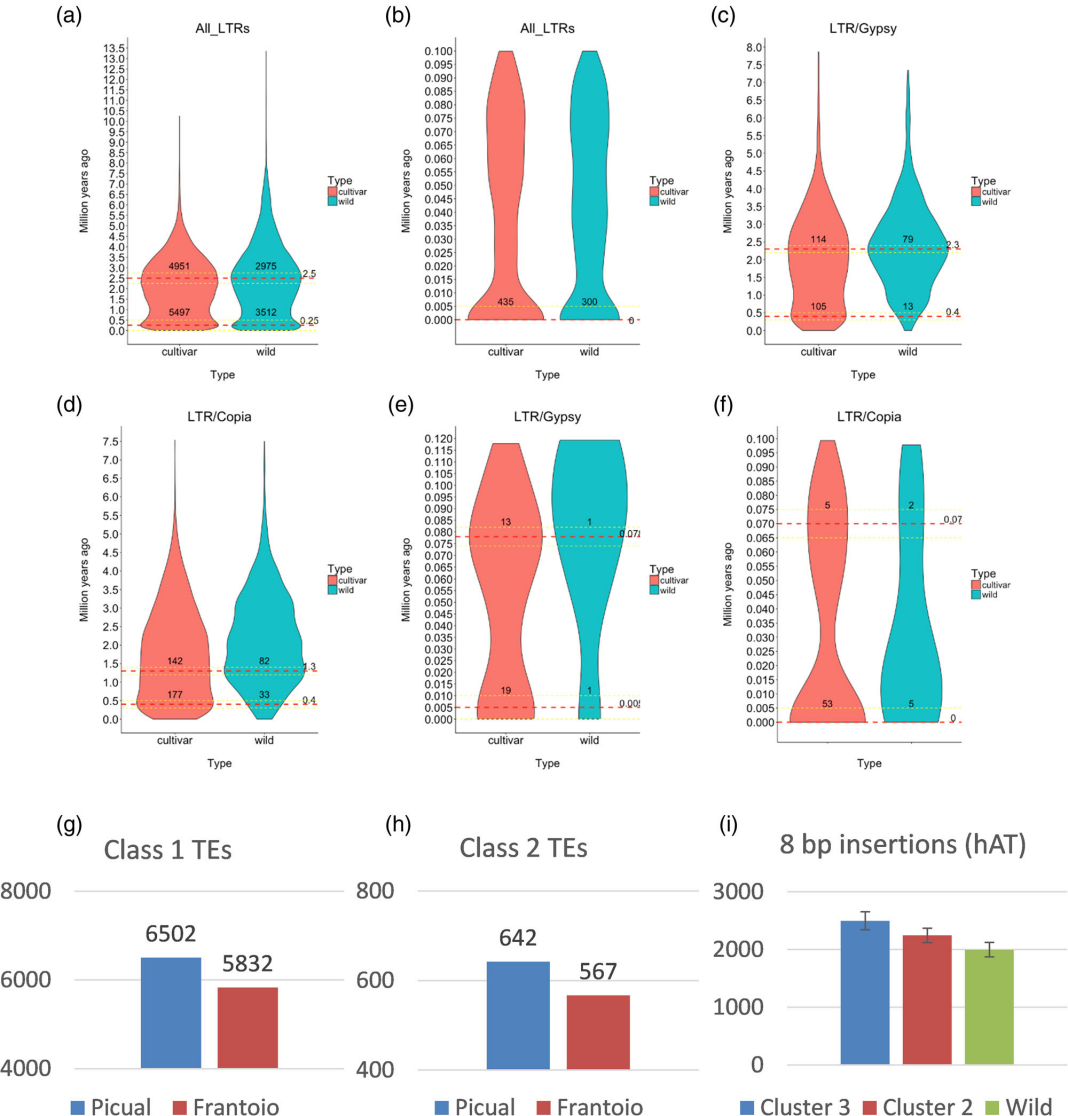
Long terminal repeat (LTR) expansion and expression analysis in cultivated and wild olive trees. (a) All LTRs expansion in the las 13 million years. (b) All LTR expansion in detail of the last 100,000 years. (c) LTR‐Gypsy expansion in the last 8 million years. (e) LTR‐Gypsy expansion in the last 100,000 years. (d) LTR‐Copia expansion in the last 7.5 million years. (f) LTR‐Copia expansion in the last 100,000 years. (g) Class 1 transposable element (TE) expressed in ‘Picual’ (Cluster 3 representative) and ‘Frantoio’ (Cluster 2 representative). (h) Class 2 TE expressed in ‘Picual’ and ‘Frantoio’. (i) The whole genome mean number of 8 bp insertions was used to estimate the Class 2 hAT TE excision activity in cultivated Cluster 2 and Cluster 3 trees and wild olives

In the first analysis, the TE landscape for the *O. europaea* var. ‘Picual’ (assembly Oleur061, representing a domesticated variety) and *O. europaea subps. sylvestris* (assembly Oe451, representing a wild type) were compared (Figure 3a–3f). In general, both genomes present similar expansion peaks around 2.5 and 0.25 MYA. A detailed analysis of recent LTR expansions during the last 5,000 years shows a higher number of LTRs in the ‘Picual’ genome (435) as compared to the wild accession (Figure 3a, 3b). In relation to the LTR‐Gypsy repeat family, a similar peak around 2.3 and 0.4 MYA can be observed in the ‘Picual’ variety, but only a single peak around 2.3 MYA is detected in the wild accession (Figure 3c). Similarly, the LTR‐Copia TE family showed different results for the ‘Picual’ variety and the wild accession. It was expanded around 1.3 MYA in the wild accession, whereas expansion was more recent in the ‘Picual’ variety (0.4 MYA). The LTR‐Copia repeats quickly shrank from the peak times to recent days in the wild accession (Figure 3d). A specific analysis of the last 5,000 years shows that the LTR‐Gypsy and LTR‐Copia families have expanded twice within 0.1 MYA in the ‘Picual’ variety. In contrast, this recent expansion is not present in the wild accession (Figure 3e, 3f). These observations may suggest that the LTR‐Gypsy and LTR‐Copia were likely activated in the ‘Picual’ cultivar during domestication. Nevertheless, these observations may be the result of a more incomplete assembly for the *O. europaea subps. sylvestris* genome (assembly Oe451) in which some TE copies may be missing or collapsed into a single copy.

In the second analysis, in order to verifying whether the TEs are transcriptionally active in the cultivated olives, RNA‐Seq data was re‐analyzed focusing on transposons. The analysis of TE expression in leaf samples of ‘Frantoio’ (Cluster 2; this work) and ‘Picual’ (Cluster 3; Leyva‐Pérez et al., 2015) showed a high transcriptional activity in both varieties, being higher the number of Class 1 and 2 TEs expressed in the Cluster 3 Picual variety (6,502 and 642, respectively) than the Cluster 2 ‘Frantoio’ variety (5,832 and 567; Figure 3g, 3h; Supplemental Table S4). Although the cut‐and‐paste mechanism of Class 2 TEs may be more difficult to detect than Class 1 insertions, they often leave a small insertion. For example, hAT TEs leave an eight‐nucleotide mark which may be detected by read mapping. The percentages of hAT TE Class 2 insertions were 25 and 12% higher in Cluster 3 (2,499 ± 158) and Cluster 2 (2,245 ± 125), respectively, than those found in wild accessions from Cluster 1 (1,997 ± 1,245; Figure 3i). This indicates that a higher TE activity may have a moderate effect in the genomic landscape of domesticated accessions.

**FIGURE 4:**
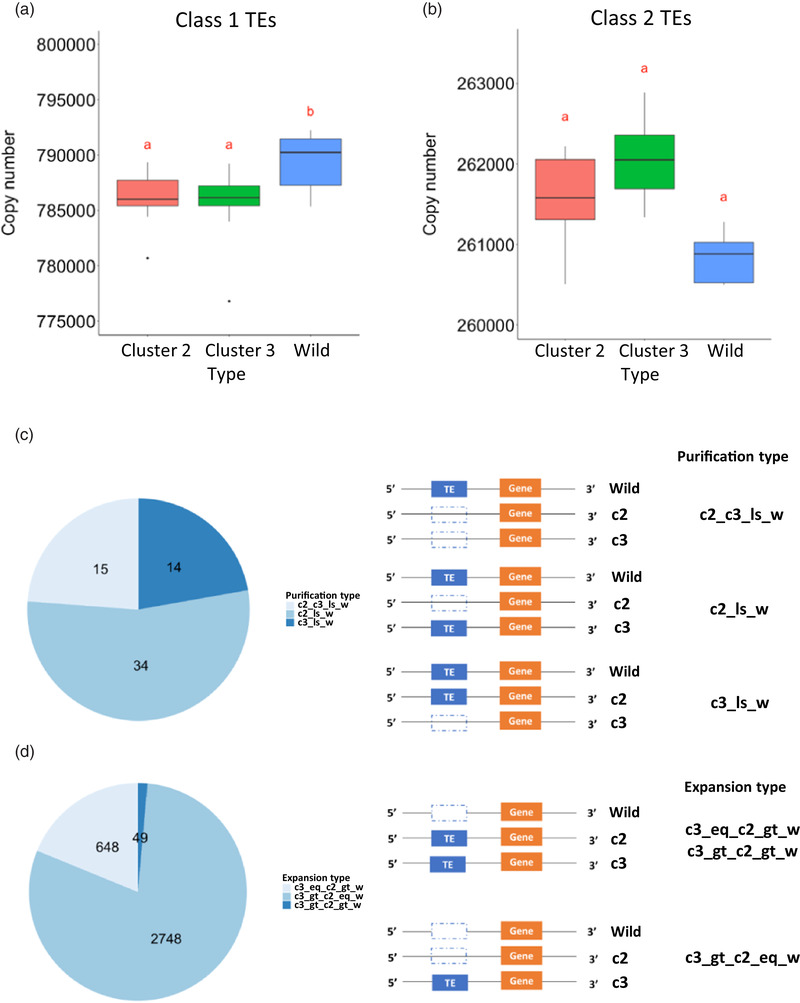
Transposable elements (TEs) copy number. Expansion and purification type TEs insertions. (a) Class 1 TEs copy number in Wild/Cluster 1 and domesticated Clusters 2 and 3 populations. (b) Class 2 TEs copy number in the same populations. (c) Purification type TE insertions. (d) Expansion type TEs insertions

In the third analysis, the TE landscape of the 51 re‐sequenced accessions were characterized in order to verify that domesticated accessions have more TEs and to estimate the possible effect of TEs in the gene space. Although the previous analysis showed that the number of LTR‐Gypsy and LTR‐Copia elements is higher in the ‘Picual’ variety than the sequenced wild accession, the TE copy number was significantly higher in wilds trees than in cultivated Clusters 2 and 3 for Class 1 TEs (Figure 4a). No difference was found in Class 2 TEs (Figure 4b). This observation suggests that although TEs may be expressed, their incorporation to the genome or purifying rate is not substantially different in domesticated accessions in comparison to wild accessions. Nevertheless, a deeper analysis of specific families revealed that 97 TE families were purified and 149 were expanded simultaneously during domestication. Namely, 3,510 individual TEs were differentially enriched among the three populations. These TE insertions were classified into: purification type, where TEs were removed out from cultivars (Figure 4c), and expansion type, where TEs were inserted into new positions in cultivars during domestication (Figure 3d). Most TE dynamics were of the expansion type rather than the purification type, and nearly 80% TEs (2,748 out of 3,445) were more likely enriched in Cluster 3 relative to Cluster 1 (Wild) and Cluster 2 populations (Figure 4c, 4d). These results may also support the idea that the Oe451 assembly may be more incomplete than the Oleur061.

To study the influence of these repetitive elements in the gene space landscape, an analysis of the nearby loci was performed among the cultivars and wild populations, specifically in the families showing purification or expansion during domestication (Supplemental Table S4). Eleven genes were located close to 13 TEs under purifying selection. Data mining on these genes revealed a Myb transcriptional activator as a possible candidate involved in abiotic stress resistance (Dubos et al., 2010; Supplemental Table S6). In line with this, 127 genes associated to the 233 expanded TEs were identified (Supplemental Table S7). Of these 127 genes, five genes had annotations related to reproductive processes, and two genes were related to embryogenesis and gametophyte development. Three of these 127 genes had putative functions related to the activation of long‐chain fatty acids, the induction of lipid transfer and lipid mobilization during seed germination, suggesting the importance of their potential role for seed oil production. Taken together, the activated TEs jumped and enriched nearby selected genes during domestication, possibly producing phenotypic diversity, for example, influencing reproductive ability in cultivated olive trees.

Domestication allows plant species to change their phenotypes, aiming to obtain traits human beings find desirable. During domestication, mutations within genes contribute to phenotypic changes (Asano et al., 2011; Azhaguvel & Komatsuda, 2007; Li & Gill, 2006). Those related to transposon amplification may have a prominent role in the regulation of gene expression, leading to phenotypic plasticity in plants during domestication. For example, an insertion of the LTR retrotransposon Dasheng in a rice granule‐bound starch synthase gene causes the glutinous trait of rice seed in the Oragamochi cultivar (Hori, Sato, & Nishio, 2007). Likewise, the unstable pigmentation patterns of maize kernels were caused by insertion of the nonautonomous DNA TE dissociation (Ds) element (Feschotte, Jiang, & Wessler, 2002). Thus, activated TEs can insert near genes to cause mutations during domestication. It can be hypothesized that, with regard to the olive tree, duplication of retrotransposons (Class 1 TEs) during domestication will be more frequent in cultivars than in wild genotypes. However, the Class 1 TE copy number of wild genotypes was significantly higher than the TE copy number in cultivars (Figure 4a). This could be explained by the fact that TEs experienced purification (97 TE families) and expansion (149 TE families) simultaneously during domestication (Supplemental Figure S3; Supplemental Table S5). Transposons can cause functional and structural modifications in chromatin, resulting in alternations of their nearby genes (Kidwell & Lisch, 1997). Eight genes surrounded by these dynamic TEs were associated with reproduction, photosynthesis, seed development and oil production (Supplemental Table S7). These genes and traits should be selected during domestication since cultivars obtained higher productive ability and seed oil production or other preferable agronomic traits than wild plants. Another set of genes nearby these TEs which were not subject to human selection were annotated to abiotic and biotic stress resistance (Supplemental Table S7). These genes might be generated by selection sweep, but further evidence should be provided. Potentially, these selected genes have produced these phenotypic diversities during domestication. The transposon dynamical insertions near the selected alleles could contribute to phenotypic diversities during olive tree domestication.

## CONFLICT OF INTEREST

The authors declare no conflict of interest.

## Supporting information

Supplemental Material

Supplemental Material

Supplemental Material

Supplemental Material

Supplemental Material

Supplemental Material

Supplemental Material

Supplemental Material

Supplemental Material
