# Supplementary material for: Transposon activation is a major driver in the genome evolution of cultivated olive trees (Olea europaea L.)
Source: Plant Genome. 2020 Mar 27;13(1):e20010. doi: 10.1002/tpg2.20010 (PMC12806974; doi:10.1002/tpg2.20010)
Supplement: Supplementary file 1 [file TPG2-13-e20010-s002.docx]

Supplemental table S3: Picual genome assembly Oleur061 covered by wild Illumina reads.

| **DNA sequences mapped** | **Length covered (%)** | **Length uncovered (%)** | **Length uncovered (bp)** | **Length uncovered – Picual (bp)** | **Uncovered genes** | **Uncovered genes –Picual** |
| --- | --- | --- | --- | --- | --- | --- |
| Picual | 97,47 | 2,53 | 42.503.421 | 0 | 5546 | 0 |
| Wild05 | 89,26 | 10,74 | 180.429.543 | 137.926.122 | 13418 | 7872 |
| Wild06 | 85,96 | 14,04 | 235.868.788 | 193.365.367 | 14841 | 9295 |
| Wild07 | 86,42 | 13,58 | 228.140.894 | 185.637.472 | 15303 | 9757 |
| Wild09 | 86,48 | 13,52 | 227.132.907 | 184.629.486 | 14386 | 8840 |
| **Mean ± SD** |  |  |  | **175.4 ± 25,3** |  | **8941 ± 805** |

150 bp paired-end reads of 4 wilds and Picual control at a depth coverage of 25-30x were mapped on the Oleur061 assembled genome.
