# Supplementary material for: Transposon activation is a major driver in the genome evolution of cultivated olive trees (Olea europaea L.)
Source: Plant Genome. 2020 Mar 27;13(1):e20010. doi: 10.1002/tpg2.20010 (PMC12806974; doi:10.1002/tpg2.20010)
Supplement: Supplementary file 2 [file TPG2-13-e20010-s003.docx]

Supplemental table S4: TE expressed in leaves of cultivated Frantoio (cluster 2) and Picual (cluster 3) trees.

| **TE** | **Frantoio**  No of TE expressed | **Picual**  No of TE expressed |
| --- | --- | --- |
|  | | |
| hAT-Ac | 106 | 118 |
| hAT-Charlie | 20 | 20 |
| hAT-Tag1 | 49 | 45 |
| hAT-Tip100 | 93 | 115 |
| hAT other | 1 | 4 |
| MULE-MuDR | 145 | 171 |
| CMC-EnSpm | 78 | 85 |
| PIF-Harbinger | 5 | 19 |
| TcMar-Pogo | 18 | 15 |
| RC/Helitron | 44 | 50 |
| Other | 8 | 0 |
| **Total number** | **567** | **642** |
|  | | |
| LINE/L1 | 378 | 311 |
| LTR/COPIA | 3,664 | 4,067 |
| LTR/Gypsy | 1,782 | 2,110 |
| LTR/Caulimovirus | 8 | 14 |
| **Total number** | **5,832** | **6,502** |

TE elements with mRNA level above 1 RPKM were considered as expressed in RNAseq leave samples. As a whole, cluster 3 was higher that cluster 2 with p value ≤ 0.05 for both, class 1 and class 2 TE.
